# Underreporting of unfavorable outcomes of congenital syphilis on the Notifiable Health Conditions Information System in the state of São Paulo, Brazil, 2007-2018

**DOI:** 10.1590/S2237-96222023000200007

**Published:** 2023-07-14

**Authors:** Larissa Festa, Marli de Fátima Prado, Amanda Cristina Santos Jesuino, Rita de Cássia Xavier Balda, Ângela Tayra, Adriana Sañudo, Mariza Vono Tancredi, Maria Aparecida da Silva, Valdir Monteiro Pinto, Daniela Testoni Costa-Nobre, Carlos Roberto Veiga Kiffer, Carla Gianna Luppi

**Affiliations:** 1 Universidade de São Paulo, Faculdade de Medicina, São Paulo, SP, Brazil; 2 Secretaria de Estado da Saúde de São Paulo, Centro de Referência e Treinamento em DST/Aids, São Paulo, SP, Brazil; 3 Universidade de São Paulo, Faculdade de Saúde Pública, São Paulo, SP, Brazil; 4 Universidade Federal de São Paulo, Escola Paulista de Medicina, São Paulo, SP, Brazil

**Keywords:** Congenital Syphilis, Health Information Systems, Epidemiological Surveillance, Infant mortality, Descriptive Epidemiology, Sífilis Congénita, Sistemas de Información Sanitaria, Monitoreo Epidemiológico, Mortalidad Infantil, Epidemiología Descriptiva, Sífilis Congênita, Sistemas de Informação em Saúde, Monitoramento Epidemiológico, Mortalidade Infantil, Epidemiologia Descritiva

## Abstract

**Objective:**

to describe the frequency of underreporting of unfavorable outcomes of congenital syphilis in the state of São Paulo, Brazil, 2007-2018.

**Methods:**

this was a descriptive study of cases of abortion, fetal and non-fetal deaths due to congenital syphilis reported on the Notifiable Health Conditions Information System (*Sistema de Informação de Agravos de Notificação* - SINAN), and those of congenital syphilis registered in any line in the Death Certificate, on the Mortality Information System (*Sistema de Informações sobre Mortalidade* - SIM), by means of probabilistic and deterministic linkage.

**Results:**

of the 27,713 cases of congenital syphilis reported, 1,320 progressed to death (871 fetal deaths, 449 infant deaths) and were matched to the SIM; 355 deaths (259 fetal deaths, 96 infant deaths) were not included on SINAN; there was an increase in unfavorable outcomes,11.4% for infant deaths due to congenital syphilis, 3.0% for fetal deaths and 1.9% for abortions.

**Conclusion:**

the use of different relationship techniques proved to be adequate to identify the frequency of underreporting of unfavorable outcomes of congenital syphilis in the state of São Paulo.

## Resumo

**Objetivo:**

descrever a frequência de subnotificação de desfechos desfavoráveis da sífilis congênita no estado de São Paulo, Brasil, 2007-2018.

**Métodos:**

estudo descritivo dos casos de aborto, óbitos fetais e não fetais por sífilis congênita notificados no Sistema de Informação de Agravos de Notificação (Sinan), e daqueles registrados com sífilis congênita, em qualquer linha da Declaração de Óbito, no Sistema de Informações sobre Mortalidade (SIM), mediante relacionamentos probabilístico e determinístico.

**Resultados:**

dos 27.713 casos de sífilis congênita notificados, 1.320 evoluíram para óbito (871 fetais, 449 infantis) e foram pareados com o SIM; 355 óbitos (259 fetais, 96 infantis) não constavam no Sinan; ocorreu incremento de desfechos desfavoráveis, de 11,4% para óbitos infantis por sífilis congênita, 3,0% para óbitos fetais e 1,9% para abortos.

**Conclusão:**

o emprego de diferentes técnicas de relacionamento mostrou-se adequado para identificar a frequência da subnotificação dos desfechos desfavoráveis da sífilis congênita no estado de São Paulo.

## Resumen

**Objetivo:**

describir la frecuencia de la subnotificación de resultados desfavorables por sífilis congénita en el estado de São Paulo, de 2007 a 2018.

**Métodos:**

estudio descriptivo de los casos de aborto espontáneo, muertes fetales y no fetales por sífilis congénita notificados en la Información Sistema de Enfermedades de Declaración Obligatoria (Sinan), y las registradas con sífilis congénita en el Sistema de Información de Mortalidad (SIM) mediante relaciones probabilísticas y determinísticas.

**Resultados:**

de los 27.713 casos de sífilis congénita, fallecieron 1.320 (871 fetales, 449 infantiles) y se emparejaron con SIM; 355 muertes (259 fetales, 96 infantiles) no se incluyeron en Sinan. Hubo un aumento en los resultados desfavorables: 11,4% muertes infantiles por sífilis congénita; 3,0% muertes fetales y 1,9% abortos.

**Conclusión:**

el uso de diferentes técnicas de vinculación demostró ser adecuado para identificar la frecuencia de subregistro de resultados desfavorables de sífilis congénita en el estado de São Paulo.

**Table:** 

Study contributions	
**Main results**	There was underreporting of unfavorable outcomes of congenital syphilis in the state of São Paulo, between 2007 and 2018. After linkage between SINAN and SIM databases, infant deaths from congenital syphilis increased by 11.4%.
**Implications for services**	The importance of the quality of data related to the outcome of congenital syphilis cases and the possibility of applying database linkage techniques in the surveillance routine can contribute to the quality of information.
**Perspectives**	Linkage between health information systems is essential to obtain more accurate estimates of the distribution and consequences of the most common compulsorily notifiable diseases and health conditions in the country.

## INTRODUCTION

Despite the efforts made to eliminate congenital syphilis and achieve the 2030 Agenda for Sustainable Development Goals, adopted by the United Nations General Assembly,^1^ congenital syphilis is still considered a leading cause of unfavorable pregnancy outcomes due to occurrence of fetal and infant deaths, abortions and low birth weight, among other serious consequences.^2^ Globally, in 2016, there were an estimated 661,000 congenital syphilis cases and more than 200,000 fetal and neonatal deaths.^2^

In Brazil, the incidence rate of congenital syphilis was 9.9 cases per 1,000 live births (LB) in 2021.^3^ That same year, of the 27,019 reported cases of congenital syphilis, 8.8% progressed to unfavorable outcomes: 1,069 fetal or infant deaths, and 1,026 abortions.^3^ In the state of São Paulo, the incidence rate of congenital syphilis increased by 184% in the period from 2011 to 2021;^3^ in 2021, the incidence rate was 7.1 cases per 1,000 LB,^3^ 14 times as high as the elimination target set by the World Health Organization (WHO),^2^ of 0.5 case per 1,000 LB.

Congenital syphilis was included in the National Compulsory Notification List of Diseases in Brazil in 1986.^4^ Congenital syphilis cases are reported on the Notifiable Health Conditions Information System (*Sistema de Informação de Agravos de Notificação* - SINAN) by filling out and typing a standardized data collection tool, the “Congenital Syphilis Case Notification/Investigation Form”, which should include information on the sociodemographic, epidemiological, clinical and evolution characteristics of the case: alive; death from congenital syphilis or other causes; fetal death; abortion.^5^ In situations in which fetal death and infant death occurred, this information should also be recorded on the Mortality Information System (*Sistema de Informações sobre Mortalidade* - SIM), given that it includes data on fetal and infant deaths that occurred in the country, and their respective causes.^6^

In order for SINAN data to reliably portray the magnitude of the disease, the epidemiological surveillance system must have quality, that is, completeness, coherence of information and absence of duplicity.^7^ Quality of data on case progression is necessary to monitor the severity of congenital syphilis, i.e., unfavorable outcomes.^2^

Studies conducted in Recife, the capital city of the state of Pernambuco,^8^ in the state of Ceará^9^ and in its capital, Fortaleza,^10^ focused on different periods in the 2010s, showed the occurrence of underreporting of congenital syphilis deaths; these results were obtained with the use of techniques for linkage between the SINAN and SIM databases. In the work routine of epidemiological surveillance teams, the automated linkage between these databases could be one of the strategies for qualifying information on the outcome of reported cases of congenital syphilis; however, this process is hampered due to the absence of an unambiguous identifier and the fragmentation of these systems.^11^ ^),(^ ^12^

Linkage between databases can be performed by means of deterministic or probabilistic methods.^13^ ^),(^ ^14^ Deterministic method uses a set of rules based on results of agreement or disagreement between matching records. This is a method that does not require the use of specific programs and complex calculations, although it has the disadvantage of requiring a common unambiguous identifier between the databases to be linked.^13^ In turn, regarding the probabilistic method, given the absence of an unambiguous identifier, the blocking of nominal variables is used in the matching. The disadvantage of the probabilistic method lies in the complexity of the process and the possible occurrence of non-matching pairs.^14^

Linkage between the SINAN and SIM congenital syphilis databases is relevant, in terms of qualifying the data on unfavorable outcomes of congenital syphilis.

The aim of this study was to describe the frequency of underreporting of unfavorable outcomes of congenital syphilis that occurred in the state of São Paulo, Brazil, from 2007 to 2018.

## METHODS

This was a descriptive study, based on notifications of cases and fetal deaths and infant deaths due to congenital syphilis on SINAN and SIM, respectively, in the state of São Paulo, between 2007 and 2018.

In the period from 2007 to 2018, the state of São Paulo registered 16.3% of the reported cases of congenital syphilis in Brazil;^3^ In 2021, it had a total population of 46,649,132 inhabitants^15^ and accounted for 31% of the national gross domestic product (GDP).^16^ That same year, in São Paulo, there were 5,027 primary healthcare centers (PHC), 777 general hospitals and an extensive health surveillance network with 356 units.^17^

Cases of congenital syphilis (i) in children under 1 year old reported on SINAN, between 2007 and 2018, were included in the study, and (ii) fetal and infant deaths recorded on SIM in the same period, under the codes A50.0 to A50.9 of the International Statistical Classification of Diseases and Related Health Problems 10^th^ Revision (ICD-10) as the underlying or associated cause of death (whether this was mentioned in any line - part I and part II)

A case of congenital syphilis was considered to be “all newborns, fetal death (stillbirth, after 22 weeks of pregnancy or weighing more than 500 grams) or abortion (pregnancy loss, up to 22 weeks of gestation or weighing less than or equal to 500 grams) of a woman with untreated or inadequately treated syphilis,” according to the Ministry of Health’s definition.^19^

The following situations were considered fetal or infant deaths due to congenital syphilis: fetal or infant death that found a matching record on the SIM database under the codes A50.0 to A50.9 of the ICD-10 as the underlying or associated cause; records on SINAN with progression to “stillbirth” or “death due to congenital syphilis” that were not found on the SIM system; records on SINAN and SIM under the codes A50.0 to A50.9 of ICD-10 as the underlying or associated cause.

We used data from the SINAN database obtained from the Epidemiological Surveillance of the São Paulo’s State Program on STD/AIDS, on July 1, 2019; and from the SIM, obtained from the Coordination for Disease Control within the São Paulo State Health Department, on March 1, 2020. In order to allow the pairing, the data were provided with the identification of the individuals.

**Figure 1:**
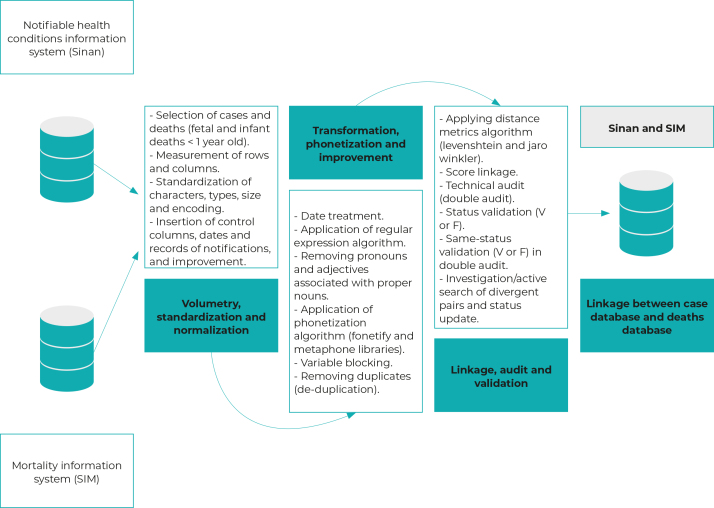
Steps of linkage between congenital syphilis case database and congenital syphilis death database

Linkage between the SINAN and SIM databases was comprised of three stages (Figure 1). In the first stage, volumetry, standardization and normalization, the procedures for preparing the database fields were performed. This process consisted of measuring rows and columns, and standardizing field categories (characters, type, size, box, and encoding).

The second stage consisted of transformation, phonetization and improvement by performing date treatment and applying a regular expression algorithm by removing terms associated with names. Phonetization consisted in converting the words into a code, so that the comparison was made by the phonetic code and not by the written word. In these procedures, the Phyton® language was used through the Fonetify and Metaphone libraries.

In addition, duplicate records were identified and removed by blocking with nominal fields, such as name of the case and mother’s name, sex and date of birth. Three subsequent steps were used in the treatment of duplicate records: identification, selection and improvement. In the case of duplicity, the record kept was the first one to enter on the database.

The third stage of procedures was related to linkage, audit and validation. For the record matching, algorithms of the Python® language were used in the R® software with the respective libraries NumPy, Pandas and Record Linkage. In this operational stage, the databases were already clean, standardized, normalized, phonetized and without duplication. For the deterministic linkage, 100% similarity between the records was used as a matching criterion. In order to retrieve the non-matching records, a probabilistic linkage^20^ was performed with a cut-off point of 90%.^21^

The audit and validation of pairs of records were performed by a pair of technical reviewers, to ensure the identification and validation of the exact matches. In case of agreement, validation was automatic, while in discordant, ambiguous, twin or homonymous cases, the complete records were reviewed and, when necessary, they were forwarded to the notifying unit for investigation.

As a final and complementary step to the pairing, an investigation and active search for information was carried out together with the regional and/or municipal epidemiological surveillance groups, in cases of congenital syphilis without any information on SINAN about their progression, as well as in the investigation of non-notified deaths. The information obtained was incorporated into the database resulting from linkage between SINAN and SIM, with the correction of case progression, when necessary.

The study variable was case progression (infant death from congenital syphilis; infant death from other causes; fetal death; abortion; ignored). Cases of congenital syphilis that progressed to death (fetal or infant death with congenital syphilis) and abortion were classified as “unfavorable outcome of congenital syphilis”.

A descriptive analysis of frequency distribution of the observed categories was performed, comparing the percentage values between the SINAN databases: original and linked databases (after linkage). The analysis was performed by means of the distribution the absolute frequency and relative frequency of congenital syphilis cases; The calculation of the percentage change of the number of cases was performed (resulting from the subtraction of the final value from the initial value, divided by the initial value, multiplied by 100).

The study project was approved by the Research Ethics Committees (REC) of the STD/AIDS Reference and Training Center [Opinion No. 4,007,885, issued on May 5, 2020; Certificate of Submission for Ethical Appraisal (*Certificado de Apresentação para Apreciação Ética* - CAAE) No. 26960919.9.0000.5375] and the São Paulo Municipal Health Department (Opinion No. 4,042,404, issued on May 22, 2020; CAAE No. 26960919.9.3001.0086), approved by both institutions.

## RESULTS

The original SINAN congenital syphilis database had 29,536 records. A total of 1,823 cases were excluded from the analysis: 72 duplicate records; 43 aged 1 year and older; and 1,708 records of occurrences outside the study period. The SIM database was comprised of 2,080 records of fetal and infant deaths due to congenital syphilis in children under 1 year old, all of them were included in the database linkage.

**Figure 2:**
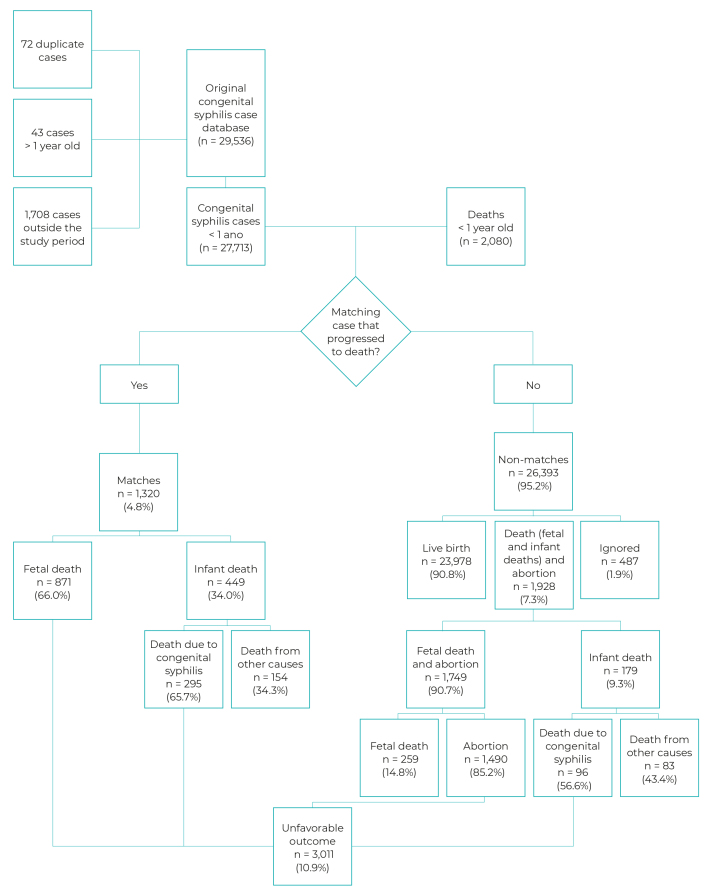
Distribution of progression of congenital syphilis cases and congenital syphilis deaths after linkage between the databases

After linking the 27,713 cases reported on SINAN to the SIM database, 1,320 (4.8%) cases were paired and 26,393 (95.2%) were non-matches. Of the 26,393 non-matching cases, 23,978 (90.8%) were recorded as live births, 1,490 (5.6%) as abortions, 487 (1.9%) as ignored, 259 (1.0%) as fetal deaths, 96 (0.4%) as infant deaths from congenital syphilis and 83 (0.3%) infant deaths from other causes. Of the 1,320 cases linked to SIM, 871 (66.0%) were fetal deaths, 295 (22.3%) infant deaths from congenital syphilis and 154 (11.7%) deaths from other causes. We identified 3,011 (10.9%) unfavorable outcomes of congenital syphilis among matching and non-matching individuals: 1,490 (49.5%) abortions, 1,130 (37.5%) fetal deaths, and 391 (13%) infant deaths from congenital syphilis (Figure 2).

The highest percentage change between the versions occurred in the progression to “infant deaths from other causes” (-11.9%) and in the progression to “infant deaths from congenital syphilis” (+11.4%). The smallest change observed was in the progression to “abortion”: 1.9%. The percentage change in unfavorable outcomes was 3.5% (Table 1).

**Table 1:** Progression of congenital syphilis cases on the Notifiable Health Conditions Information System databases (SINAN), original and linked databases, state of São Paulo, Brazil, 2007-2018

Progression of congenital syphilis cases	SINAN database				Percentage change	
	Original database		Linked database			
	N	%	N	%	n	%
Infant deaths with congenital syphilis	351	12.1	391	13.0	40	11.4
Fetal deaths	1,097	37.7	1,130	37.5	33	3.0
Abortion	1,462	50.2	1,490	49.5	28	1.9
Subtotal (unfavorable outcomes)	2,910	100.0	3,011	100.0	101	3.5
Infant deaths or other causes of death	269	8.5	237	7.3	-32	-11.9
Total	3,179	100.0	3,248	100.0	69	2.2

## DISCUSSION

There was underreporting of unfavorable outcomes of congenital syphilis on SINAN in the state of São Paulo. The highest underreporting occurred in the outcome “infant death from congenital syphilis”. The database linkage process was able to identify underreporting of unfavorable outcomes.

When comparing with the data from the Epidemiological Bulletin in the state of São Paulo, it could be seen underreporting of fetal and infant deaths from congenital syphilis found in the state, given that, in the same period, 299 infant deaths with congenital syphilis were reported; that is, a 30.7% increase was observed through the linkage process adopted.^5^ This difference is greater when compared with data obtained from the SIM database: for the same period, 178 deaths with congenital syphilis were reported, that is, a difference of 119%.^22^

The underreporting of deaths due to congenital syphilis has already been the subject of cross-sectional studies conducted using the SIM and SINAN databases in other states^8^ ^)-(^ ^10^ that applied similar techniques for linkage between cases and deaths. In Recife,^8^ in the period from 2010 to 2016, the proportion of underreporting of fetal and infant deaths from congenital syphilis on SINAN was 80.9%; in the state of Ceará,^9^ ^)^ in the period from 2010 to 2014, this proportion was 89.4%; and in Fortaleza, in the period from 2007 to 2013, it was 90.1%.^10^

The difference observed regarding the number of fetal deaths from congenital syphilis was smaller when compared to the number of infant deaths. A possible explanation for this finding would be the low quality of filling in the cause of death field in fetal deaths on the SIM system, which are more frequently recorded as resulting from unspecified causes.^23^ Moreover, there may also be underestimation of fetal deaths that, despite the efforts made by the death surveillance teams, still occur in Brazil, and they are higher than those found in developed countries.^24^

A slight underreporting of abortion cases was observed by means of this linkage method. This result was expected, especially when taken into consideration that the event is not recorded on the SIM database, the source used for linking it to the SINAN database in this study. The difference attributed was due to the result of the active search investigation, by which the regional surveillance teams found cases with incorrect filling in of the “progression” field in the SINAN investigation/notification form. This finding was also described in the investigation conducted in the state of Ceará, where linkage between the SINAN and SIM databases was used.^9^

Underreporting of unfavorable outcomes, especially those regarding fetal deaths and deaths in children under 1 year of age, should not be so high, since fetal and infant deaths are systematically investigated in Brazil by means of the infant and fetal death surveillance.^24^ The results of this study showed that, despite the occurrence of surveillance on a continuous basis, it was still necessary to carry out an active search for a new investigation of cases of congenital syphilis, regarding their outcomes. Therefore, aiming to reduce the underreporting of congenital syphilis, it is necessary to review the application of the criteria used for classification of causes of death by epidemiological surveillance teams and infant and fetal death prevention committees.^8^

In this study, “death from congenital syphilis” was considered to be one in which congenital syphilis was included in any line of the death certificate (DC). A study conducted in the same period, in the Metropolitan Region of São Paulo, adopting this same criterion for the analysis of infant deaths due to congenital syphilis, showed a 97% increase in the outcome “death”, when compared to that found through the analysis of the SIM database.^25^

Linkage between the databases performed with the combination of two linkage techniques - deterministic and probabilistic - contributed to the success of data matching. The use of probabilistic approach provides retrieval of records of the same individual, which were not identified in the deterministic approach.^27^ Thus, using hybrid record linkage shows better results when compared to techniques performed independently, a methodology adopted in the implementation of linkage records in the Colorado Congenital Heart Disease surveillance system, United States, with individuals aged 11 to 64 years, registered in the system as having congenital heart disease, between 2011 and 2013.^27^

Some limitations of this study should be presented. A limiting factor for the database linkage was lack of an unambiguous and common identifier key, which resulted in operational difficulties and demanded the use of probabilistic techniques for the identification of pairs based on character strings, such as mother’s name and/or child’s name (on the SINAN databases, most cases were still identified with the term newborn or stillborn preceding their mother’s name). In addition, the occurrence of failures in the standardization of the filling out of identification fields used for database linkage, errors in the registration or coding of causes of death and the long time elapsed between the occurrence of the outcome and the investigation may have led to the occurrence of false matches. To minimize the possibility of false matches, a case validation strategy was used, performed by a pair of independent investigators.

This study identified underreporting of unfavorable outcomes of congenital syphilis in the state of São Paulo. Aiming to contribute to the reduction of this underreporting, the application of database linkage techniques proved to be adequate, a practice that can be incorporated into the health surveillance routine as a tool for improving information and monitoring compulsorily notifiable diseases and health conditions, including congenital syphilis.
